# Long-term effects of human induced pluripotent stem cell-derived retinal cell transplantation in *Pde6b* knockout rats

**DOI:** 10.1038/s12276-021-00588-w

**Published:** 2021-04-08

**Authors:** Jee Myung Yang, Sunho Chung, KyungA Yun, Bora Kim, Seongjun So, Seoon Kang, Eunju Kang, Joo Yong Lee

**Affiliations:** 1 Department of Ophthalmology, Asan Medical Center, University of Ulsan College of Medicine, 88 Olympic-ro 43-gil, Songpa-gu, Seoul, 05505 Republic of Korea; 2 Stem Cell Center, Asan Institute for Life Sciences, Asan Medical Center, University of Ulsan College of Medicine, Seoul, Republic of Korea; 3 Department of Convergence Medicine, Asan Institute for Life Sciences, Asan Medical Center, University of Ulsan College of Medicine, Seoul, Republic of Korea

**Keywords:** Translational research, Stem-cell research, Retina

## Abstract

Retinal degenerative disorders, including age-related macular degeneration and retinitis pigmentosa (RP), are characterized by the irreversible loss of photoreceptor cells and retinal pigment epithelial (RPE) cells; however, the long-term effect of implanting both human induced pluripotent stem cell (hiPSC)-derived RPE and photoreceptor for retinal regeneration has not yet been investigated. In this study, we evaluated the long-term effects of hiPSC-derived RPE and photoreceptor cell transplantation in *Pde6b* knockout rats to study RP; cells were injected into the subretinal space of the right eyes of rats before the appearance of signs of retinal degeneration at 2–3 weeks of age. Ten months after transplantation, we evaluated the cells using fundus photography, optical coherence tomography, and histological evaluation, and no abnormal cell proliferation was observed. A relatively large number of transplanted cells persisted during the first 4 months; subsequently, the number of these cells decreased gradually. Notably, immunohistochemical analysis revealed that the hiPSC-derived retinal cells showed characteristics of both RPE cells and photoreceptors of human origin after transplantation. Functional analysis of vision by scotopic electroretinogram revealed significant preservation of vision after transplantation. Our study suggests that the transplantation of hiPSC-derived retinal cells, including RPE cells and photoreceptors, has a potential therapeutic effect against irreversible retinal degenerative diseases.

## Retinal disease: Cell transplants offer hope

Cells with the potential to regenerate retinal cells could become a useful treatment for serious eye diseases such as age-related macular degeneration and retinitis pigmentosa. The retina has only limited self-regenerating potential. Joo Yong Lee and colleagues at the University of Ulsan in Seoul, South Korea, studied the long-term effects of implanting cells derived from human stem cells into the eyes of rats that had been genetically modified to act as a model of retinal degenerative disease. The implanted cells have the features of the photoreceptor cells that detect light and those cells that generate a layer of the retina called the retinal pigment epithelium. A substantial population of the cells persisted for at least four months, significantly preserving the animals’ vision. The results justify further exploration of the therapeutic possibilities.

## Introduction

The retina is neural tissue located in the posterior part of the eye and is an extension of the central nervous system (CNS), which has limited regenerative potential once damaged^1^. Therefore, to maintain homeostasis of the retinal microenvironment and protect itself from harmful stimuli, the retina has a unique structure consisting of inner and outer blood-retinal barriers (BRBs)^2–4^. The outer BRB is mainly composed of retinal pigment epithelial (RPE) cells, which support photoreceptor cells, the primary neurons in the retina, and play a significant role in the pathogenesis of retinal degenerative disorders, such as age-related macular degeneration (AMD) and retinitis pigmentosa (RP)^5–9^. These disorders are commonly characterized by the irreversible loss of photoreceptor cells and RPE cells, and the only fundamental treatment for these retinal degenerative disorders is replacement of damaged or atrophied cells^10–12^. Thus, regenerative treatments, such as stem cell transplantation, are emerging as attractive options for targeting retinal degeneration that was previously considered untreatable^13^.

RP refers to a set of hereditary retinal degenerative disorders that initially involve photoreceptors and leads to subsequent RPE cell damage; it affects 1 in 4000 individuals worldwide^9^. Due to its inherent nature, extensive genetic studies are ongoing, and more than 50 causal genes have been identified^14^. Among the causal genes, *PDE6B* is a gene that encodes rod cGMP-phosphodiesterase, which is a critical component of the biochemical light transduction pathway^9^. Although various molecular and genetic studies have identified the pathomechanisms of RP, attempts to restore vision in patients with RP have failed. To overcome this issue, preclinical stem cell-based studies involving transient dosing or permanent implantation of pluripotent stem cells are being conducted^10,11,15,16^.

Permanent implantation of retinal stem cells is a promising method and is highly expected to be a potential alternative treatment strategy for replacing damaged retinal cells^13,16^. Sharma et al.^17^ manufactured clinical-grade AMD patient stem cell-derived RPE cells using RPE patches of a biodegradable scaffold, and functionally validated the effects of their transplantation. This researchers provided a pipeline for the generation of clinical-grade induced pluripotent stem cell (iPSC)-derived RPE cells, and histologically and functionally validated the efficacy of transplantation, thereby significantly advancing the retinal stem cell transplantation field; however, a single RPE cell transplantation cannot rescue already compromised photoreceptor cells and can be only applied in early stages of retinal degenerative diseases, when there are sufficient functional photoreceptor cells. On the other hand, the replacement of only photoreceptors has a low success rate, as the implanted cells may not be supported by healthy RPE cells. Therefore, it is ideal to transplant cells with the ability to differentiate into both RPE and photoreceptors into the degenerated retina^16^. To our knowledge, no studies have succeeded in implanting both iPSC-derived RPE cells and photoreceptors as a long-term treatment.

In this study, we demonstrated for the first time that human iPSC (hiPSC)-derived retinal cells that can differentiate into both photoreceptors and RPE cells can efficiently rescue the degenerated host retina. Using a *Pde6b* knockout rat model, which is a novel model of RP established using CRISPR-Cpf1 technology^18^, we found that transplanted hiPSC-derived retinal cells can survive in the host retina for a long time and restore retinal function.

## Materials and methods

### Animals

All animals were treated in accordance with the guidelines defined by the Association for Research in Vision and Ophthalmology Statement for the Use of Animals in Ophthalmology and Vision Research, and were bred and managed according to the Animal Experiment guidelines of the Asan Medical Center. Our experimental methods were reviewed and approved by the Institutional Animal Care and Use Committee (approval number 2017-12-169).

*Pde6b* knockout rats and SD rats (OrientBio, Gyeonggi, Korea) were housed in a specific-pathogen-free environment on a 12 h/12 h light–dark cycle at the Asan Medical Center. In the present study, hiPSC-derived retinal cells were transplanted into *Pde6b* knockout rats at the age of 2–3 weeks. hiPSC-derived retinal cells were subretinally injected into the right eye, whereas vehicle (phosphate-buffered saline; PBS) was injected into the left eye as an untreated control. The rats were euthanized 2 weeks, 2 months, or 4 months after transplantation, and the eyeballs were removed for analysis.

### Differentiation of hiPSCs into retinal cells

To induce differentiation of hiPSCs into retinal cells, hiPSCs were plated at a density of 2 × 10^4^/cm^2^ in vitronectin-coated 12-well plates and cultured until 80% confluency was achieved. Then, the cells were cultured for 14 days in neurobasal induction medium; the induction medium was replaced every day to initiate RPE and photoreceptor cell differentiation. The neurobasal induction medium consisted of Dulbecco’s modified Eagle’s medium (DMEM) supplemented with F12, 1× N2, 1× B27, and 1× nonessential amino acids (Life Technologies), and the following cytokines at different stages of the differentiation process: differentiation day 0–1: 10 mM nicotinamide (Sigma), 50 ng/ml noggin, 10 ng/ml DKK-1, and 10 ng/ml insulin-like growth factor-1 (IGF-1; Peprotech); differentiation days 2–3: 10 mM nicotinamide, 10 ng/ml noggin, 10 ng/ml DKK-1, 10 ng/ml IGF-1, and 5 ng/ml FGF2; differentiation days 4–5: 10 ng/ml DKK-1, 10 ng/ml IGF-1, and 100 ng/ml activin A (Peprotech); differentiation days 6–7: 100 ng/ml activin A, and 10 µM SU5402 (Sigma); and differentiation days 8–14: 100 ng/ml activin A, 10 µM SU5402, and 3 µM CHIR99021 (Peprotech). Fasudil (10 µM, Adooq), a ROCK inhibitor, was added to the medium on all differentiation days^19^.

At the end of the 14-day RPE and photoreceptor cell differentiation process, the cells had reached a density of ~1 × 10^5^ cells/cm^2^ in vitronectin-coated dishes. On day 14, the cells were passaged using Accutase (Gibco) and cultured in X-Vivo medium (Lonza) supplemented with 10 µM fasudil to support the enrichment, and expansion of the RPE and photoreceptor cells. The medium was replaced every three days after each subculture step.

### Characterization of hiPSC-derived retinal cells by immunocytochemistry

Differentiated cells were fixed with 4% formaldehyde overnight at 4 °C and washed twice with phosphate-buffered saline (PBS). The cells were permeabilized and blocked through incubation in blocking solution comprising 1% BSA, 0.1% Triton X-100, and 0.1% Tween 20 in PBS for 30 min at room temperature. After this, the cells were incubated overnight at 4 °C with primary antibodies against melanocyte-inducing transcription factor (MITF; Abcam, 1:200) and CHX10 (Santa Cruz, 1:200), which are RPE cell and neural retina markers, respectively. The primary antibodies were then removed by washing with 0.1% Tween 20 in PBS, and the cells were incubated at room temperature for 1.5 h with the following secondary antibodies: goat anti-rabbit IgG H&L (1:500, Alexa Fluor 555-conjugated, Abcam) for MITF and goat anti-mouse IgG H&L (1:1000, Alexa Fluor 488-conjugated, Abcam) for CHX10. After antibody incubation was completed, the nuclei were counterstained by incubation with 100 ng/ml 4′,6-diamidino-2-phenylindole (DAPI, Sigma) in PBS for 30 min at room temperature. All fluorescence images were acquired using a fluorescence microscope (AxioObserver Z1; Carl Zeiss, Oberkochen, Germany).

### Subretinal transplantation

A sclerotomy was initially made using a sterile 26-gauge needle (Video 1). Then, 5 µl of an hiPSC-derived retinal cell suspension with a concentration of 7.4 × 10^5^ cells/μl was injected into the subretinal space of the right eyes of *Pde6b* knockout rats, using a Hamilton syringe with a 33-gauge needle through the previously made sclerotomy. PBS served as a control and was injected into the left eye. The cells were washed with PBS before transplantation and dissociated to allow the release of individual cells into X-Vivo medium. *Pde6b* knockout rats (aged 2–3 weeks) were anesthetized by intraperitoneal administration of a mixture of 0.4 ml/kg xylazine hydrochloride, 0.6 ml/kg tiletamine hydrochloride, and zolazepam hydrochloride. The pupils were dilated using tropicamide and phenylephrine hydrochloride eye drops (Mydrin-P; Santen Pharmaceutical Co., Ltd., Osaka, Japan). Subretinal injection was performed under a surgical microscope (Carl Zeiss). After conjunctival peritomy, a 26-gauge needle was used to inject the cells. Then, using a Hamilton syringe with a 33-gauge needle, 5 μl hiPSC-derived retinal cells were injected into the subretinal space. After the injection, hypromellose (Hycell solution 2%; Samil Pharmaceutical Co., Ltd., Seoul, Korea) was applied to prevent drying of the eye, and an infrared lamp was used to maintain body temperature.

### OCT imaging

Retinal cross-sectional images were obtained 2 weeks, 1 month, 2 months, 5 months, and 10 months after transplantation, using optical coherence tomography (OCT; IIS Science, Korea). Using B-scan OCT angiography, we obtained angiographic images of the retinal layers and retinal surfaces. These images were obtained using the same protocols used for anesthesia administration and pupillary dilation. During OCT imaging, hypromellose was applied to both eyes to prevent excessive drying of the ocular surface during recovery. The animals were placed on a heating pad, and the eye of interest was positioned directly in front of the scan head lens.

### ERG recording

The rats were housed in the dark for >12 h before electroretinogram (ERG) recording was performed, and only dim red light was used for preparation in a darkroom. Anesthesia administration and pupillary dilation were performed using the same protocols as those used in the transplantation procedure. For OCT imaging, hypromellose was topically applied to prevent drying of the eyes. The rats were placed on a heating pad to maintain body temperature. Two electrode needle tips were inserted between the eyes, and another was inserted under the tail skin. A gold lens was then placed near the cornea to measure the electrical signals from the retina. A Granzfeld stimulator was used to deliver a total of three to ten green flashes, and the results were averaged for analysis. Flashes of 1.3 log cd/m^2^ green light were used for scotopic ERG with a sampling rate of 1000 Hz and bandpass filter of 2.5 mV. After recording, infrared light was used to maintain body temperature until the animals regained consciousness.

### Human mtDNA validation using conventional PCR and Sanger sequencing

Retinal tissue samples containing hiPSC-derived retinal cells were harvested 2 weeks and 4 months after transplantation, and paraffin-embedded sections (10 µm thick) were observed under an Olympus CX41 microscope (Olympus America, Center Valley, PA, USA). Mitochondrial DNA (mtDNA) was performed using a PicoPure® DNA Extraction Kit (Applied Biosystems). PCR master mix (AccuPower® PCR Master Mix) and primers (F-5′-GCCTTCCCCCGTAAATGATA-3′ and R-5′-CTTCTGTGGAACGAGGGTTT-3′; mtDNA 15 S rRNA) were used to detect transplanted mtDNA of human origin. The final PCR volume was 20 µl, which included 10 µl PCR master mix, 1 µl DNA template, 7 µl nuclease-free water, and 1 µl each of the forward and reverse primers. The DNA template concentrations were 500 and 5000 ng/µl for the samples harvested 2 weeks and 4 months after transplantation, respectively. The positive and negative controls were hiPSC mtDNA and mtDNA obtained from the retinas of *Pde6b* knockout rats, respectively. PCR was performed under the following thermal cycling conditions: initial denaturation at 95 °C for 1 min; 35 cycles of 95 °C for 15 s, 56 °C for 15 s, and 68 °C for 1 min; and a final elongation at 68 °C for 3 min. The amplified samples were then electrophoresed at 100 V on a 2% agarose gel and visualized using a GelDoc XR+ Imaging System (Bio-Rad).

### Hematoxylin and eosin staining

Two weeks, 2 months, and 4 months after transplantation, the animals were euthanized using a CO_2_ chamber, and the eyeballs were harvested. Enucleated eyes were fixed in Davison’s buffer for 6 h and embedded in 4% formalin solution overnight. Vertical sections with a thickness of 4 µm were prepared, mounted on slides, stained with hematoxylin and eosin (H&E), and viewed under an Olympus CX41 microscope (Olympus America, Center Valley, PA, USA).

### Immunohistochemical analysis of retinal tissue

Deparaffinization was performed by washing the slides twice with 100% ethanol; then, the slides were rehydrated by washing with 95, 80, and 75% ethanol. The slides were then placed in 1× antigen retrieval solution (10× citrate buffer, ImmunoBioscience, #AR-6544-05) and boiled in a pressure cooker (Instant Pot Duo Multicooker; Instant Brands Inc., Canada) for 30 min at 120 °C and 10 min at 90 °C. After cooling the slides to room temperature, blocking was performed using 5% goat serum (normal goat serum, S-1000; Vector Laboratories) and 0.1% Triton in PBS. The slides were incubated with the appropriate primary antibodies overnight at 4 °C, and subsequently with secondary antibody for 1 h at room temperature. The slides were mounted using mounting solution with DAPI (VECTASHIELD® Hardset Antifade Mounting Medium, H-1400; Vector Laboratories).

Human TRA-1-85/CD147 (MAB3195; Millipore, 1:100 dilution), MITF (AB122982; Abcam, 1:100 dilution), Recoverin (AB5585; Millipore, 1:100 dilution), PDE6B (sc-377486; Santa Cruz Biotechnology, 1:100 dilution), and CD45 (AB10558; Abcam, 1:100 dilution) primary antibodies were used to label human cells, RPE cells, photoreceptor cells, and inflammatory cells, respectively. DyLight 594-conjugated (DI-1594; Vector Laboratories, 1:1000) and FITC-conjugated (A-11029; Invitrogen, 1:1000) secondary antibodies were used. Images were taken using a Zeiss LSM 880 upright confocal microscope (Carl Zeiss, Germany). DAPI staining was used to identify and count cells in the retinal area of interest. Costaining with TRA-1-85/CD147 was performed to identify cells of human origin^20^.

### Statistical analyses

Statistical analysis was performed using GraphPad Prism 7.0. Normal distribution was verified using the Shapiro–Wilk test. Statistical data are presented as the mean ± standard error of the mean, and *p* values were determined using the Mann–Whitney *U* test and unpaired two-tailed Student’s *t* test.

## Results

### Differentiation of hiPSCs into retinal cells

**Fig. 1:**
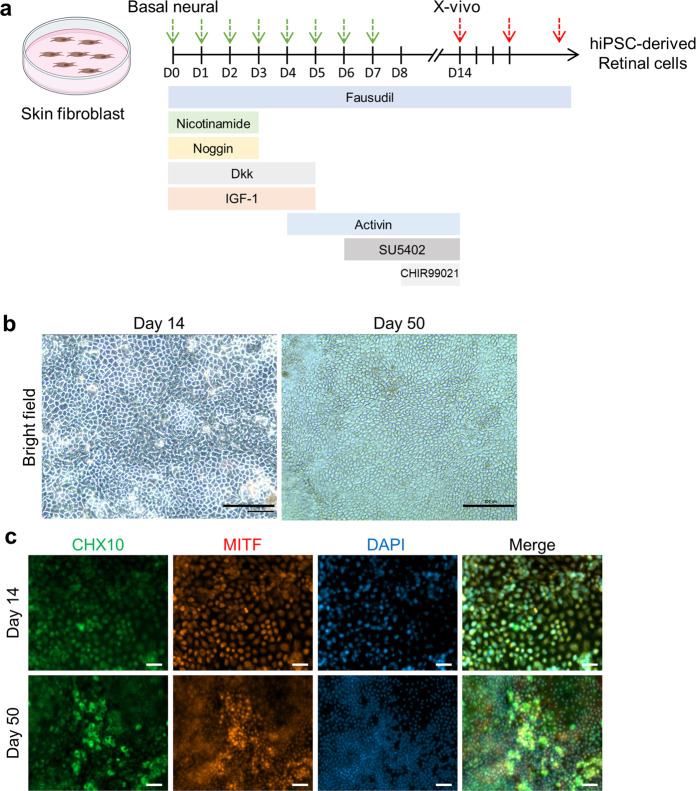
Characterization of hiPSC-derived retinal cells. **a** Schematic diagram of the differentiation of hiPSC-derived retinal cells (created with BioRender). **b** Light microscopy images of retinal cells derived from hiPSCs 14 and 50 days after differentiation. **c** Immunocytochemical analysis of the RPE cell marker MITF and the photoreceptor marker CHX10 (VSX2) in hiPSC-derived retinal cells 14 and 50 days after differentiation. Both MITF and CHX10 (VSX2) were expressed in hiPSC-derived retinal cells. DAPI was used to stain the nuclei. Scale bars, 50 μm.

The ability of hiPSCs derived from skin fibroblasts to differentiate into retinal cells was verified in vitro (Fig. 1a). Microscopic examination of the phenotype of hiPSC-derived retinal cells 14 and 50 days after differentiation revealed that the cells exhibited the same shape as differentiated RPE cells and contained brown pigment (Fig. 1b). The expression of the RPE cell marker MITF and the photoreceptor marker CHX10 (VSX2) was measured 14 and 50 days after the differentiation of hiPSCs (Fig. 1c). MITF and CHX10 (VSX2) expression overlapped, indicating that hiPSC-derived retinal cells concurrently exhibited both RPE cell and photoreceptor characteristics.

### Transplantation of hiPSC-derived retinal cells into *Pde6b* knockout rats

**Fig. 2:**
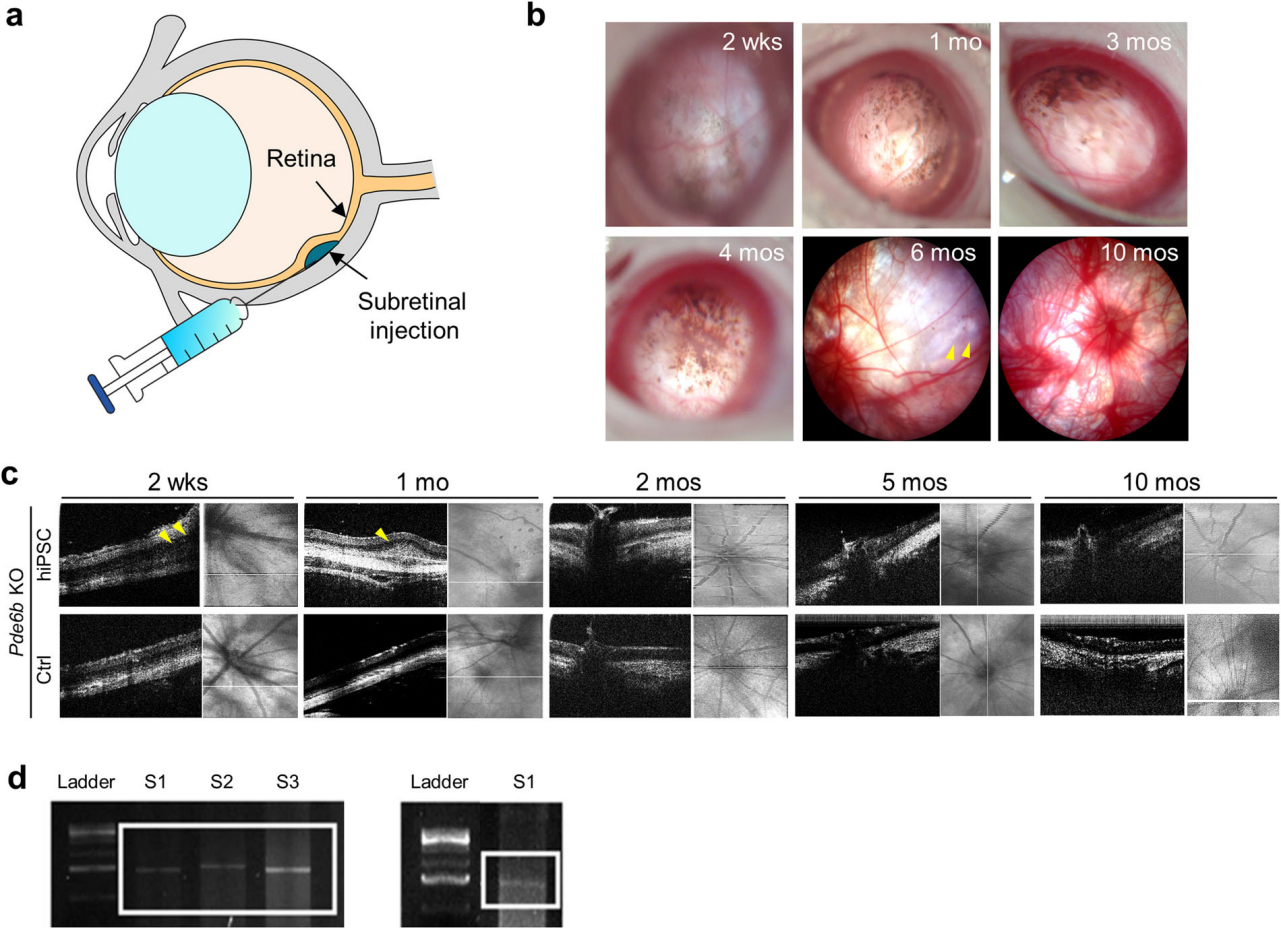
Morphological changes in the retinas of *Pde6b* knockout rats after the transplantation of hiPSC-derived retinal cells into the subretinal space. **a** Schematic diagram of subretinal injection of hiPSC-derived retinal cells. **b** Fundus images of the transplantation area taken between 2 weeks and 10 months after the injection of cells. The yellow arrowheads indicate the transplantation area. **c** Optical coherence tomography (OCT) images taken between 2 weeks and 10 months after transplantation. The yellow arrowheads indicate the transplantation area. The white line on each fundus image indicates the location of the OCT scan. **d** The results of the PCR analysis of mitochondrial DNA confirmed the presence of hiPSC-derived retinal cells at the injection site 2 weeks (DNA template concentration of 500 ng/μl) and 4 months (DNA template concentration of 5000 ng/μl) after transplantation. M1: 1 kb ladder. Left lanes 1, 2, 3: 2 weeks; right lane 1: 4 months. hiPSC-R hiPSC-derived retinal cell transplantation group. Scale bars, 100 μm.

Differentiated hiPSCs with the characteristics of both RPE cells and photoreceptors were transplanted into the subretinal space by injection (Fig. 2a and Video 1). Afterward, serial color fundus photographs were obtained to verify retinal cell transplantation. From 2 weeks to 10 months after injection, the presence of hiPSC-derived retinal cells was monitored according to the presence of brown pigment, which is a typical characteristic of retinal cells (Fig. 2b). We observed that the hiPSC-derived retinal cells injected into the subretinal space were maintained for up to 6 months and that incessant proliferation was not observed. Brown pigment was most apparent 2 weeks after transplantation, but faded and was not observable after 10 months.

OCT imaging of the right eye (treated) and the left (control) eye was periodically performed for 10 months after transplantation (Fig. 2c). Two weeks and 1 month after transplantation, OCT images indicated that the injected cells were located in the subretinal space. OCT imaging revealed a decrease in total retinal thickness, which is a characteristic of retinal degeneration; however, there was an increase in total retinal thickness at the injection area. No significant difference in retinal thickness was observed around the optic nerve.

To verify that the cells observed at the injection site were of human origin, we performed mtDNA analysis. Conventional PCR of mtDNA obtained from paraffin-embedded sections of tissue taken 2 weeks and 4 months after transplantation was performed (Fig. 2d). The subretinal transplantation cell area was identified by microscopy; by analyzing tissue taken 2 weeks (DNA template concentration of 500 ng/μl) and 4 months (DNA template concentration of 5000 ng/μl) after transplantation using PCR, the presence of cells of human origin in the injection area was confirmed.

**Fig. 3:**
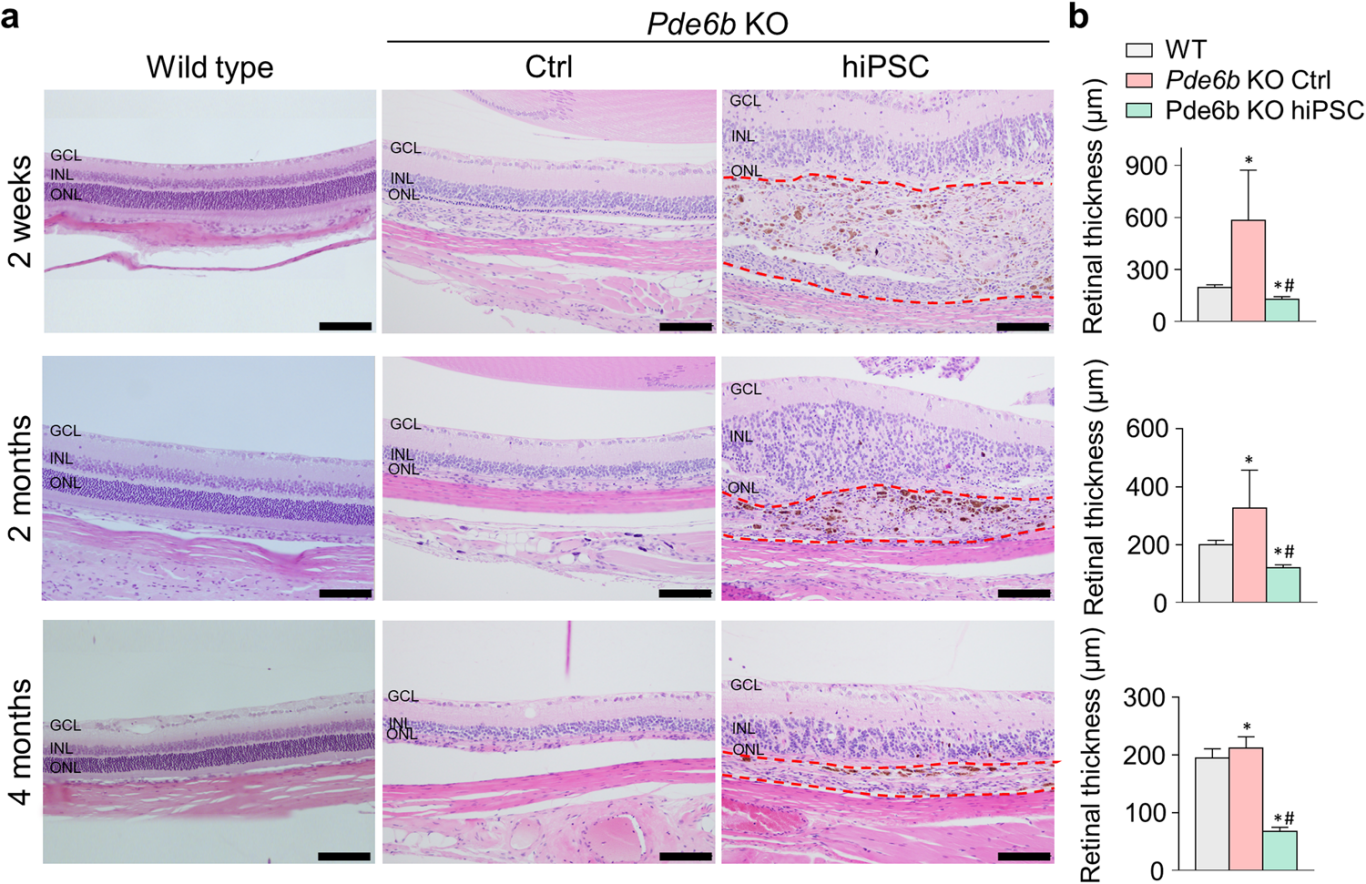
H&E staining of the retinas of wild-type Sprague–Dawley rats, untreated *Pde6b* knockout rats, and *Pde6b* knockout rats transplanted with hiPSC-derived retinal cells. **a** H&E staining was conducted 2 weeks, 2 months, and 4 months after hiPSC-derived retinal cell transplantation. Sprague–Dawley (SD) rats of the same age as the experimental animals were used for comparison at each time point after transplantation. Pigment was apparent within the transplantation area. In treated *Pde6b* rats, the red dotted line indicates the transplantation area. **b** Quantification of the retinal thicknesses. GCL ganglion cell layer, INL inner nuclear layer, ONL outer nuclear layer. Scale bars, 100 μm. *p < 0.05 compared to wild-type rats, ^#^*p* < 0.05 compared to *Pde6b* KO controls (untreated).

The morphological characteristics of the retina, including the photoreceptor layer and subretinal space, in both the control and treated eyes of *Pde6b* knockout rats 2 weeks, 2 months, and 4 months after hiPSC-derived retinal cell transplantation were histologically analyzed (Fig. 3a). We generated a *Pde6b* knockout rat model as a retinal degeneration model that typically shows generalized retinal cell damage and reduced retinal thickness 3 weeks after postnatal development^18^. Cells were injected before the expected appearance of retinal damage at 2–3 weeks of age to decelerate retinal degeneration, and support the recovery of RPE cell and photoreceptor function.

Two weeks after transplantation, the total retinal thickness at the transplantation site (584.8 ± 288.2 μm) was 4.5 times greater than the total retinal thickness in the untreated eyes (128.6 ± 15 μm; Fig. 3b). Two months after transplantation, the total retinal thickness in the treated eyes (325.3 ± 132.1 μm) was 2.7 times greater than that in the untreated eyes (119.9 ± 10.1 μm). Four months after transplantation, the total retinal thickness in the treated eyes (211.2 ± 19.9 μm) was 3.1 times greater than that in the untreated eyes (67.3 ± 7.1 μm). Up to 4 months after transplantation, the total retinal thickness in the treated eyes remained significantly greater than that in the untreated eyes and remained stable, suggesting a delay in retinal degeneration.

### Histological analysis of hiPSC-derived retinal cells transplanted into *Pde6b* knockout rats

**Fig. 4:**
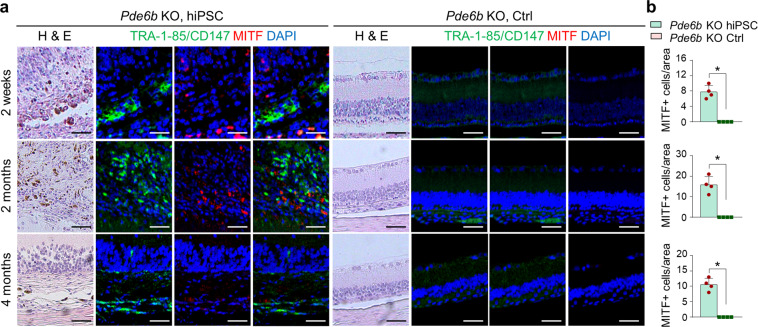
RPE cells in the area in which hiPSC-derived retinal cells were injected. **a** Images of H&E staining and immunohistochemical analysis of the same area in the retinas of *Pde6b* knockout rats 2 weeks, 2 months, and 4 months after transplantation. **b** Quantification of RPE cells following transplantation (*n* = 4 retinas per group). Red: MITF, a specific marker of rat and human RPE cells. Green: TRA-1-85/CD147, a human-specific marker. Blue: DAPI, a nuclear marker. *Pde6b* KO *Pde6b* knockout. Scale bars, 50 μm. *p < 0.05.

**Fig. 5:**
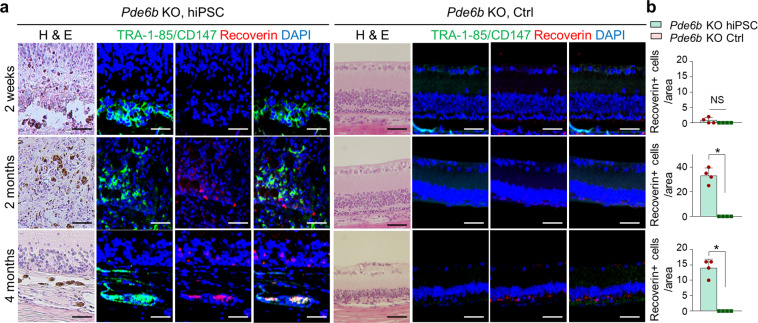
Photoreceptors in the area in which hiPSC-derived retinal cells were injected. **a** Images of H&E staining and immunohistochemical analysis of the same area in the retinas of *Pde6b* knockout rats 2 weeks, 2 months, and 4 months after transplantation. **b** Quantification of photoreceptor cells following transplantation (*n* = 4 retinas per group). Red: Recoverin, a marker of photoreceptor cells. Green: TRA-1-85/CD147, a human-specific marker. Blue: DAPI, a nuclear marker. *Pde6b* KO *Pde6b* knockout. Scale bars, 50 μm. *p < 0.05.

**Fig. 6:**
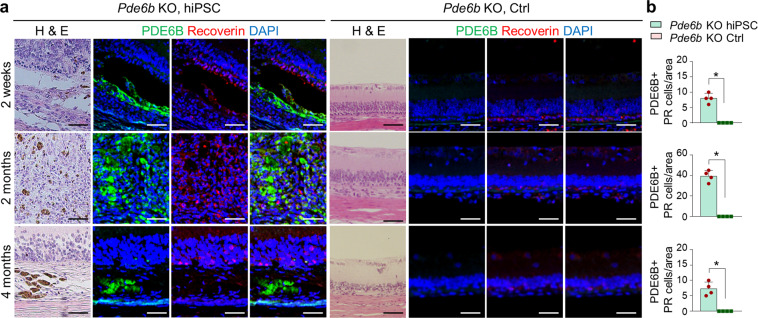
PDE6B expression in the area in which hiPSC-derived retinal cells were injected. **a** Images of H&E staining and immunohistochemical analysis of the same area in the retinas of *Pde6b* knockout rats 2 weeks, 2 months, and 4 months after transplantation. **b** Quantification of PDE6B expression following transplantation (*n* = 4 retinas per group). Red: Recoverin, a marker of photoreceptor cells. Green: PDE6B, a *Pde6b* gene marker. Blue: DAPI, a nuclear marker. *Pde6b* ko *Pde6b* knockout. Scale bars, 50 μm. *p < 0.05.

Histological analysis revealed a significantly greater retinal thicknesses in the treated eyes than the untreated eyes. The presence of transplanted cells was subsequently confirmed by conventional PCR analysis. Cells were transplanted into the subretinal space in *Pde6b* knockout rats at the age of 2–3 weeks, and immunohistochemistry was performed to confirm that they were maintained in vivo (Figs. 4, 5, and 6). Staining for the human-specific marker TRA-1-85/CD147 confirmed the presence of human iPSC-derived cells. In addition, colocalization of TRA-1-85/CD147 with the RPE marker MITF (Fig. 4), and the human- and rat-specific photoreceptor marker Recoverin (Fig. 5) was observed for up to 4 months in vivo. Colocalization of the *Pde6b* gene product PDE6B and Recoverin confirmed the presence of the PDE6B protein in the subretinal space in *Pde6b* knockout rats, suggesting the recovery of photoreceptors in hiPSC-treated rats (Fig. 6). The transplanted cells exerted a protective effect by maintaining endogenous photoreceptor cells in the areas where TRA-1-85/CD147 and Recoverin were not colocalized; a greater degree of Recoverin staining was observed in the treated eyes than in the untreated eyes. MITF expression at 4 months after transplantation was markedly lower than that at 2 weeks. In addition, the number of transplanted RPE cells, which were identified as cells coexpressing MITF and TRA-1-85/CD147, was lower at 4 months than at 2 weeks after transplantation. Two weeks after transplantation, MITF expression was higher than Recoverin expression; however, the expression of these two markers was similar beginning at 2 months. Both RPE cells and photoreceptor cells were found to persist for up to 4 months after transplantation, and the expression of the relevant cell-specific markers was higher in the eyes treated with hiPSC-derived cells than in the control eyes. Furthermore, PDE6B expression was observed in the eyes treated with hiPSC-derived cells 2 weeks, 2 months, and 4 months after transplantation; however, PDE6B expression was not detected in the control eyes. The above results indicate that after the transplantation of hiPSC-derived retinal cells in vivo, RPE cells are initially observed in higher numbers than photoreceptors and may differentiate into cells with the properties of photoreceptors.

### Functional analysis of *Pde6b* knockout rats after hiPSC-derived retinal cell transplantation

**Fig. 7:**
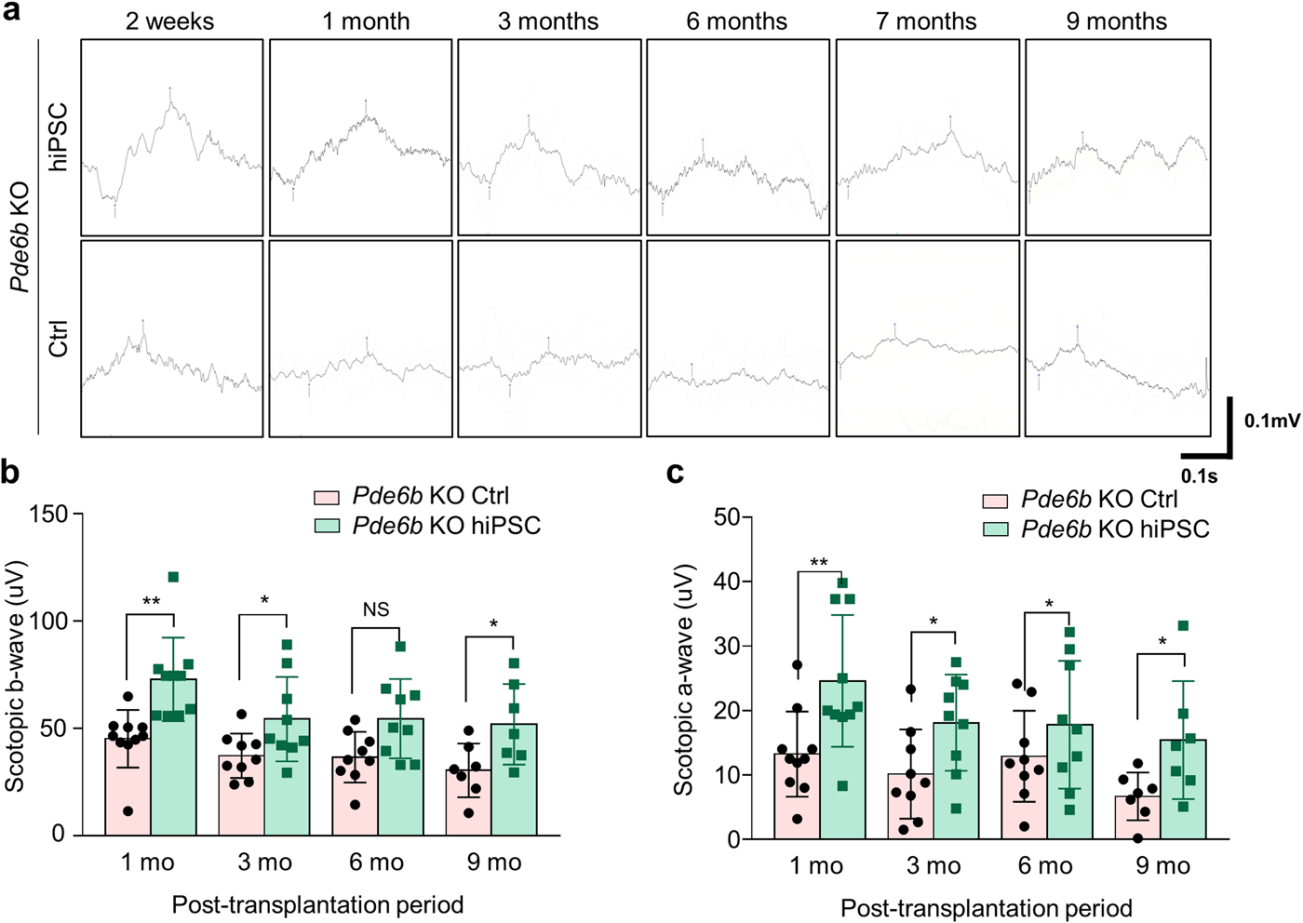
hiPSC-derived retinal cell transplantation preserved photoreceptor function in *Pde6b* knockout rats. **a** Representative ERG responses recorded between 2 weeks and 9 months after transplantation. **b**, **c** Bar charts showing the mean scotopic b-wave and a-wave amplitudes elicited by green light with an intensity of 1.3 log cd/m^2^. The results showed that under dark conditions, the ERG b-wave (**b**) and a-wave (**c**) amplitudes were significantly higher in the group treated with hiPSC-derived retinal cells than in the control group. hiPSC-R hiPSC-derived retinal cell transplantation group. Number of eyes: *n* = 10 at 1 month, *n* = 9 at 3 months, *n* = 9 at 6 months, and *n* = 6 at 9 months. Error bars: standard error of the mean. *p < 0.05, **p < 0.01.

Scotopic ERG was used to assess the treated and control eyes of *Pde6b* knockout rats 2 weeks to 9 months after transplantation. Waveforms gradually of both the treated and untreated eyes of *Pde6b* knockout rats flattened over this time period. In the treated eyes, the shape of the waveform was retained from 3 weeks after transplantation until 9 months after transplantation (Fig. 7a). Two weeks after transplantation, when the *Pde6b* knockout rats were 4–5 weeks old, their corresponding waveforms were comparable to those of a normal SD rat of the same age. In *Pde6b* knockout rats, ERG responses were approximately three times lower than those in normal SD rats.

Quantified rod-related ERG responses indicated that the b-wave amplitude was significantly higher in the treated eyes than in the untreated eyes of *Pde6b* knockout rats (72.9 ± 6.1 vs. 45.3 ± 4.2 μV, *p* = 0.002) 1 month after transplantation (Fig. 7b). Furthermore, the b-wave amplitude at 3 months after transplantation was significantly higher in the treated eyes than in the untreated eyes (54.4 ± 6.6 vs. 37.3 ± 3.5 μV, *p* = 0.03). The b-wave amplitude at 6 months after transplantation was also significantly higher in the treated eyes than in the untreated eyes (54.5 ± 6.1 vs. 36.7 ± 3.9 μV, *p* = 0.02). The b-wave amplitude gradually decreased from 1 month to 9 months after transplantation in the treated eyes; however, the b-wave amplitude at 9 months after transplantation remained significantly higher in the treated eyes than in the untreated eyes (55.7 ± 7.2 vs. 30.5 ± 5.6 μV, *p* = 0.02) and reached a period of stabilization. Further analysis of quantified rod-related ERG responses revealed that the a-wave amplitude was significantly higher in the treated eyes than in the control eyes (24.6 ± 3.2 vs. 13.3 ± 2.1 μV, *p* = 0.008) 1 month after transplantation (Fig. 7c). The a-wave amplitude 3 months after transplantation was also significantly higher in the treated eyes than in the untreated eyes (18.1 ± 2.5 vs. 9.8 ± 2.5 μV, *p* = 0.03). Six months after transplantation, there was no significant difference in the a-wave amplitude between the treated and untreated eyes of *Pde6b* knockout rats (17.8 ± 3.3 vs. 12.5 ± 2.6 μV, *p* = 0.22). The a-wave amplitude gradually decreased from 1 month to 9 months after transplantation in the treated eyes, a period of stabilization was reached at 9 months posttransplantation, and the a-wave amplitude remained significantly higher in the treated eyes than in the untreated eyes of *Pde6b* knockout rats (17.1 ± 3.6 vs. 7.8 ± 1.1 μV, *p* = 0.03).

## Discussion

In this study, we transplanted hiPSC-derived retinal cells into the subretinal space in *Pde6b* knockout rats, and examined the effect of the transplanted RPE and photoreceptor cells on retinal degeneration over a long period of times. We successfully differentiated hiPSC-derived retinal cells into both RPE and photoreceptor cells, and confirmed the effects of the transplanted hiPSC-derived retinal cells by examining intraocular changes over time via ocular imaging and histological evaluation. The transplanted hiPSC-derived retinal cells were stable for 4 months after transplantation. Then, the level of pigmentation, which is indicative of the transplanted cells in the subretinal space, started to decrease, and this decrease persisted for 10 months. Transplanted hiPSC-derived retinal cells also delayed photoreceptor degeneration and the functional decline of vision in *Pde6* knockout rats.

AMD and RP are retinal diseases that eventually lead to the loss of photoreceptor cells^7,9,15^. Studies involving transplantation of stem cell-derived photoreceptor cells or RPE cells have shown that these cells exert a protective effect by slowing the rate of damage in progressive retinal diseases^10,16^. While published studies on the differentiation of hiPSCs into RPE cells are available, there are no reports in which transplanted hiPSC-derived retinal cells simultaneously show the characteristics of photoreceptor cells and RPE cells^16^. The hiPSC-derived retinal cells used for transplantation in our study exhibited the characteristics of both photoreceptor cells and RPE cells. In a study involving transplantation of mouse iPSC-derived retinal ganglion cells and photoreceptors, the expression of pluripotent genes was decreased in neurally induced cells compared with control cells, and neural progenitors showed the potential to form neural networks^21^. Furthermore, in transplanted hiPSC-derived RPE cells, the presence of cells in transplantation areas was confirmed, and functional improvements were identified in the treated group compared to the untreated group; however, no recent studies have studied transplanted cells showing the characteristics of both RPE cells and photoreceptors. In genetic retinal diseases and AMD, which are caused by photoreceptor and RPE damage, visual function cannot be preserved by transplanting differentiated RPE cells alone, as these diseases result in damage to the whole retina and not just RPE cells, photoreceptors or choroid capillaries^8,22^. Thus, it is expected that the transplantation of retinal cells with characteristics of both photoreceptors and RPE cells would be more effective in preserving visual function. Further studies are needed to verify that the retinal cells used in this study can form neural networks.

Unlike other retinal diseases in which treatment is administered after the development and progression of the disease, the goal of RP treatment is early intervention to delay retinal damage before it becomes severe since the damage is irreversible^9^. In addition, the development of imaging techniques had made early detection of RP possible, and therapeutic strategies for this subset of patients aim to delay disease progression through early intervention^15^. Therefore, based on clinical trends and the results of our previous study^18^, we transplanted cells before the anatomical advancement of retinal degeneration.

Our study verified in various ways that the pigmentation observed under the retina after transplantation of hiPSC-derived retinal cells originated from the transplantation of human cells. To confirm that the mtDNA in cells in the transplantation area was of human origin, samples of retinal tissue containing transplanted cells were acquired, and conventional PCR was used to demonstrate that human mtDNA was present in the retinal tissue 2 weeks and 4 months after transplantation. Furthermore, immunohistochemistry revealed that the RPE marker MITF, the photoreceptor marker Recoverin and PDE6B protein were expressed at the transplantation site, and we confirmed that transplanted cells survived under the retinas of *Pde6b* knockout rats by confirming coexpression with the human-specific marker TRA-1-85. PDE6B was expressed at the transplantation site, and the presence of PDE6B protein in the subretinal space in *Pde6b* knockout rats was confirmed. Furthermore, the coexpression of PDE6B and the photoreceptor marker Recoverin was also confirmed. ERG was performed at several time points after transplantation to determine the effect of transplanted cells on vision loss due to retinal cell degeneration in the *Pde6b* knockout rats. Over the 9-month period after transplantation, the amplitude of the ERG response continually decreased in both the transplant group and the control group; however, at 1, 3, 6, and 9 months, the a- and b-wave amplitudes were higher in the treated group than in the untreated group, suggesting preservation of visual function. Considering that the area of the retina in which transplanted hiPSC-derived retinal cells were present was relatively small, it is expected that additional preservation of visual function can be achieved if cell transplantation can be achieved in a larger area in the retina. Behavioral visual tests in animals, such as optokinetic tests, are needed to demonstrate the effect of hiPSC-derived RPE cells on visual function.

iPSCs can induce retinal cell differentiation, bypass the ethical problems and immunological challenges associated with embryonic stem cells (ESCs), and can be autologously generated from patient tissue; however, whether iPSCs have the potential to form teratomas, which is the case for undifferentiated cells, remains a concern^23^. Therefore, it is important to effectively induce the differentiation of iPSCs into appropriate cell types. Transplantation of retinal cells originating from stem cells, such as ESCs and iPSCs, has shown promising results in nonclinical and clinical trials, which have found the risk of teratoma development to be low^24^. In nonclinical studies, Mandai et al. showed that mouse ESC/iPSC-derived retinal grafts can form an outer nuclear layer composed of mature photoreceptors and stably form a synaptic connection with a bipolar cell of the host^25,26^. In 2012, the first trial was performed in which hESC-derived RPE cells were injected into human patients with AMD^27^. No teratoma development or abnormal proliferation was found during the 4-month observation period, and the cells integrated stably into the RPE layer. Our study did not reveal the presence of a network of retinal nerve cells in the region of transplantation or the linear stratification of mature retinal cells, but no abnormal cell proliferation or morphological change in the subretinal space was observed in the 10 months after transplantation. In addition, the cells transplanted into the retina showed a gradual decrease in growth after 4 months, and ERG revealed an improvement in a- and b-wave amplitudes and preservation of visual function in the treated eyes compared to the untreated eyes.

The anatomical and functional survival time of the hiPSC-induced RPE and photoreceptor cells was 6–9 months in our study, which is short for use as a lifelong treatment. Previous studies have shown that the anatomical and functional survival of transplanted cells varies among species and conditions; additional, long-term results are absent from many studies, and transplanted cells generally fail to survive or induce visual restoration^16^. In immunodeficient IL-2 receptor-γ knockout mice, hESC-derived cells survived, and visual function was restored for 9 months, whereas immunocompetent mice showed preservation of the integrated cells for 3–6 months^20,28^. In patients with AMD, hiPSC-derived RPE cells retained their functions up to 1 year after transplantation^29^, and other reports have shown that improvements in visual function can be observed between 2 and 6 months after transplantation of human retinal progenitor cells, and last for a maximum of 12 months in patients with advanced RP^12,30^. Although there is a limitation in terms of comparing the age of rats to that of humans (6 months of age in rat days is estimated to be 15–20 years of age in human^31^) or members of other species, the transplanted cells did not survive for a shorter period of time in our study (6–9 months) than in previous reports.

It was previously difficult to assess the exact reason for the change in the number of transplanted retinal cells; however, immunohistochemistry conducted 2 weeks after transplantation revealed a number of inflammatory cells showing positive staining for CD45 in close proximity to cells transplanted into the subretinal space (data not shown). Although the subretinal space is considered immune-privileged^32^, the survival and functional integration of transplanted retinal cells into this area can be improved by immunosuppression^20^. Damage to transplanted cells by the inflammatory response exerted by these immune cells is thought to affect the survival of hiPSC-derived retinal cells after transplantation. Further research is needed to determine whether posttransplant immunosuppression treatment increases the survival of transplanted cells by reducing the inflammatory response. In addition to modulation of the immune response, retinal cells with better quality and purity, and the utilization of efficient transplantation techniques (e.g., sheets, biodegradable patches, etc.) are needed to improve the integration and survival of transplanted cells^33^.

A limitation of the hiPSC-derived retinal cell transplantation strategy used in our study is that the cells were delivered in suspension rather than as a cell sheet, and thus did not retain the normal laminated structure of the retina. Observations over time revealed that the hiPSC-derived retinal cells delivered in suspension existed in a mixed form after transplantation, with several cells persisting in the subretinal space, and the formation of a normal retinal structure could not be confirmed. Although retinal cells were maintained in an incomplete structure, ERG showed that visual function was better preserved in the hiPSC-treated group than the untreated group. Transplantation of retinal cells in a cell sheet has been performed in studies on medium to large animals and in clinical human studies^10^. In animal models of retinal degeneration that received cell sheet transplantation, the damage to both the existing retinal cell layer and the transplanted tissue was low. In fact, Hu et al. showed that after injection of an ultrathin substrate containing a monolayer of hESC-derived RPE cells, <2% of transplanted cells were lost, which is a suitable level of cell loss for transplantation^34^. Another study suggested that posttransplantation cell survival is greater for polarized cells than suspended cells^35^. The form and method of transplantation used in our study were different, but we found a similar improvement in visual function and preservation of retinal thickness and the RPE monolayer at the injection site. In future studies, it will be helpful to develop effective differentiation methods for retinal cells to support the development of hiPSC-derived retinal cell sheets or cell patches that are more similar to normal anatomical structures, and to develop a safe transplantation method and investigate whether visual improvement and cell restoration can be improved by these transplantation methods.

In conclusion, our study showed that transplanted hiPSC-derived retinal cells showing the characteristics of both RPE cells and photoreceptors exhibit long-term survival under the host retina, and retain the characteristics of both cell types in *Pde6b* knockout rats. Furthermore, hiPSC-derived retinal cells preserve visual function in the long term by inhibiting retinal degeneration. Utilization of a more effective transplantation method, such as cell sheets or cell patches of hiPSC-derived retinal cells, may help to further preserve the anatomy and function of the degenerated retina.

## Supplementary information

Video 1. Subretinal injection of hiPSC-derived retinal cells and verification of retinal cell transplantation (pigmented area in the retina) by live fundus imaging.

Video 1 Caption
