# Supplementary material for: Long-term effects of human induced pluripotent stem cell-derived retinal cell transplantation in Pde6b knockout rats
Source: Exp Mol Med. 2021 Apr 8;53(4):631–42. doi: 10.1038/s12276-021-00588-w (PMC8102536; doi:10.1038/s12276-021-00588-w)
Supplement: Supplementary file 2 — Video 1 Caption [file 12276_2021_588_MOESM2_ESM.docx]

**Video 1.** **Subretinal injection of hiPSC-derived retinal cells and verification of retinal cell transplantation (pigmented area in the retina) by live fundus imaging.** (0–4 seconds) A sclerotomy was initially made using a sterile 26-gauge needle, (5–10 seconds) after which a 33-gauge Hamilton needle was inserted into the subretinal space through the previously made sclerotomy, and hiPSC-derived retinal cells were injected. (11–22 seconds) Live fundus imaging showing the areas in which retinal cells were transplanted.
